# Feature-based classification of human transcription factors into hypothetical sub-classes related to regulatory function

**DOI:** 10.1186/s12859-016-1349-2

**Published:** 2016-11-14

**Authors:** Rezvan Ehsani, Shahram Bahrami, Finn Drabløs

**Affiliations:** 1 Department of Cancer Research and Molecular Medicine, Norwegian University of Science and Technology, PO Box 8905, NO-7491 Trondheim, Norway; 2 Department of Mathematics, University of Zabol, Zabol, Iran; 3 St. Olavs Hospital, Trondheim University Hospital, NO-7006 Trondheim, Norway

**Keywords:** Transcription factors, Chromatin opening, Machine learning, Classification

## Abstract

**Background:**

Transcription factors are key proteins in the regulation of gene transcription. An important step in this process is the opening of chromatin in order to make genomic regions available for transcription. Data on DNase I hypersensitivity has previously been used to label a subset of transcription factors as Pioneers, Settlers and Migrants to describe their potential role in this process. These labels represent an interesting hypothesis on gene regulation and possibly a useful approach for data analysis, and therefore we wanted to expand the set of labeled transcription factors to include as many known factors as possible. We have used a well-annotated dataset of 1175 transcription factors as input to supervised machine learning methods, using the subset with previously assigned labels as training set. We then used the final classifier to label the additional transcription factors according to their potential role as Pioneers, Settlers and Migrants. The full set of labeled transcription factors was used to investigate associated properties and functions of each class, including an analysis of interaction data for transcription factors based on DNA co-binding and protein-protein interactions. We also used the assigned labels to analyze a previously published set of gene lists associated with a time course experiment on cell differentiation.

**Results:**

The analysis showed that the classification of transcription factors with respect to their potential role in chromatin opening largely was determined by how they bind to DNA. Each subclass of transcription factors was enriched for properties that seemed to characterize the subclass relative to its role in gene regulation, with very general functions for Pioneers, whereas Migrants to a larger extent were associated with specific processes. Further analysis showed that the expanded classification is a useful resource for analyzing other datasets on transcription factors with respect to their potential role in gene regulation. The analysis of transcription factor interaction data showed complementary differences between the subclasses, where transcription factors labeled as Pioneers often interact with other transcription factors through DNA co-binding, whereas Migrants to a larger extent use protein-protein interactions. The analysis of time course data on cell differentiation indicated a shift in the regulatory program associated with Pioneer-like transcription factors during differentiation.

**Conclusions:**

The expanded classification is an interesting resource for analyzing data on gene regulation, as illustrated here on transcription factor interaction data and data from a time course experiment. The potential regulatory function of transcription factors seems largely to be determined by how they bind DNA, but is also influenced by how they interact with each other through cooperativity and protein-protein interactions.

**Electronic supplementary material:**

The online version of this article (doi:10.1186/s12859-016-1349-2) contains supplementary material, which is available to authorized users.

## Background

Cells recognize and respond to internal and external signals, often leading to changes in the transcription level of specific genes. Transcriptional regulatory systems play a key role in many biological processes, such as cell cycle progression, maintenance of intracellular metabolism, physiological balance, and cellular differentiation in developmental time courses [1, 2]. The regulatory system for transcription involves several proteins, and in particular transcription factors (TFs) can coordinate a diversity of regulatory processes. Many diseases arise from errors in the regulatory system for transcription; TFs are overrepresented among oncogenes [3], and a third of human developmental disorders have been related to dysfunctional TFs [4]. However, alterations in the activity and regulatory pathway of TFs are also likely to be a source for phenotypic diversity and evolutionary adaptation [5–7].

Most TFs bind to DNA by recognizing specific DNA sub-sequences known as transcription factor binding sites (TFBSs), and thereby they control the transcription of nearby genes through their promoters, or more distant genes through enhancers. However, it has been realized that binding of TFs to TFBSs is not enough to fully explain the regulatory program of gene expression [8]. The set of cis-regulatory regions (promoters, enhancers) is identical at the DNA sequence level in all cell types of a given species. Therefore the transcriptional program specific to each cell type must be the result of the set of TFs expressed in that cell type, and how genes are selected for transcriptional activation or repression. The same TFs can be expressed at the same rate in different cell types, but may have separate binding sites, as TF function and regulatory pathways also depend upon chromatin structure and epigenetic modifications [9].

TFs can be classified according to regulatory function. Sherwood et al. (2014) introduced PIQ (protein interaction quantitation), a computational method for modeling the magnitude and shape of genome-wide DNase I hypersensitivity profiles used for identification of transcription factor binding sites [10]. They identified binding sites for more than 700 transcription factors from an experiment using DNase I hypersensitivity analysis followed by sequencing, and used the data for a hypothetical classification of transcription factors into three groups; Pioneers, Settlers and Migrants. Pioneer TFs are assumed to be distinguished by their ability to bind to DNA target sites, even in inaccessible regions, and were found bound to chromatin before activation of enhancers and gene expression modulation. The binding of Settler TFs seems to depend upon the openness of chromatin at their binding sites. They almost always bind to sites matching their DNA-binding motif, but they do not enable binding to inaccessible DNA sites [10, 11]. And finally Migrant TFs only bind to a subset of their target sites, even in accessible DNA [10].

TFs can also be classified based on structural properties, and the most common classifications are based on the structure of their DNA-binding domains (DBDs) [12]. In some instances the structural classification may also indicate the function of TFs. For example, TFs with homeodomain are often associated with developmental processes, and those with a “winged” helix-turn-helix (HTH) motif are frequently associated with the interferon regulatory factor family and triggering of immune responses against viral infections [12]. Wingender et al. (2013) have made a comprehensive classification of 1558 human TFs based on a hierarchy of general topology (Superclass), similar structures of the DBD (Class), sequence and functional similarities (Family), sequence-based subgroupings (Subfamily), TF gene (Genus), and TF polypeptide (Factor ‘species’), and this classification is known as TFClass. TFs are classified according to this six-level classification scheme, where four levels are abstractions according to different criteria, while the fifth level shows the TF genes, and the sixth level individual gene products. They collected and curated 71 animal TF families. Altogether, ten superclasses have been identified, comprising of 40 classes and 111 families [13].

In a previous paper we presented a comprehensive list of properties for 1978 human TFs. We identified 1225 DNA binding TFs, based on existing annotation of Pfam domains and identification of additional Pfam DBDs. For the remaining 753 TFs we could not identify a clear DBD. Annotated properties included DBDs, protein–protein interactions, and post-translational modifications. The paper demonstrated how such a resource can be used to identify properties that are enriched in a subset of TFs [14]. However, it is an interesting question whether TF properties also can be used to predict the regulatory function of TFs, for example as defined by Sherwood et al. This is particularly relevant as Sherwood et al. could assign regulatory function only to a subset of the known TFs.

There are several different types of classifiers from machine learning that may be used for this type of function prediction, as for example Random Forest (RF), Support Vector Classifier (SVC) with different kernels (e.g. linear, Radial Basis Function (RBF), polynomial), *k* Nearest Neighbours (kNN), and Gaussian naïve Bayes (GNB) classifiers. There are also several approaches for improving classifier performance, like boosting, where an ensemble of possibly weak classifiers as combined into a stronger classifier, and AdaBoost [15] is an important implementation of boosting.

Random Forest (RF) is one of the most successful ensemble techniques in machine learning and bioinformatics for high-dimensional classification, and also the main classifier for this project. The RF algorithm makes a large number of individual decision tree classifiers (i.e. a forest) where each tree gives a classification, and the final classification is based on the votes over all the trees in the forest. The rule to generate a tree is through splits at each node based on the yes and no answer of the predictors. The split selection can be performed by using decrease of Gini impurity in each step, where Gini impurity is a measure of how often a randomly chosen element from the set would be incorrectly labeled if it was randomly labeled according to the distribution of labels in the subset.

The goal of this project was to predict regulatory function according to Sherwood et al. for the majority of the TFs. We therefore made a training set of TFs with regulatory function as hypothesized by Sherwood et al., and used TF properties, including the structural classification by Wingender et al., to define a feature vector for each TF. We used machine learning techniques to evaluate and select classifiers and feature vectors [16–19], and showed that the structure of the DBD is the most important property for predicting regulatory function. We then used this to predict regulatory function for the TFs not classified by Sherwood et al., and analyzed the outcome of the classification. We also used the full set of classified TFs (measured and predicted) to identify properties of the functional classes by enrichment analysis, and to analyze data on TF-TF interactions as well as time course data on cell differentiation.

## Methods

### Data for training and classification

We used the comprehensive collection of properties for 1978 TFs from our previous work [14]. This included information on DNA binding domains (DBDs), protein–protein interactions (PPIs), and post-translational modifications (PTMs). The information on DNA binding domains was based on Pfam annotation and literature, plus a DBD prediction method for identification of additional DNA-binding Pfam-domains [20, 21]. The original list of 1978 human transcription factors was taken from Ravasi et al., where they generated experimental data on PPIs to build an atlas of combinatorial regulation [22], and this information on PPIs was included in our data set. We also used information about PTMs from Phosphosite [23]. For this project we extended the initial annotation by adding data on TF classification from the set of 1558 TFs classified by Wingender et al. [13] (TFClass), and 1175 TFs were found in the overlap between TFClass and our set of annotated TFs.

We then used the set of TFs classified according to chromatin activity (regulatory function) by Sherwood et al. [10]. We could identify 459 of these TFs in our database, and 457 of these had intersection with the 1175 TFs with TFClass annotation. These 457 TFs included 45 TFs with function as Pioneers, 47 as Settlers, and 365 as Migrants, and were used as training set for machine learning, which subsequently was used to classify the remaining 718 TFs from the set of 1175 TFs.

### Encoding TF properties in feature vectors

We encoded a large set of available properties in feature vectors for TF classification: TFClass, frequent Pfam domains, DNA binding (yes/no), number of DBDs, PPI (yes/no), number of PPIs, PTMs (general and individual), number of zinc fingers, and number of positions for phosphorylation. Since the properties initially were a mixture of quantitative and qualitative features (e.g. having a specific Pfam domain (yes/no) versus the number of phosphorylation sites (0, 1, 2, …)), they were converted to a more consistent binary representation before analysis, as described below and in Table 1.

**Table 1:** Summary of property encodings for transcription factors (TFs)

Property	Description	Encoding
TF_Class	Encoded 2–3 top levels of five digit code based on TFClass classification; i.e. superclass followed by class (see text)	A 47-dimensional vector where i^th^ position (superclass) and j^th^ position (class) are 1, other positions are 0
PD	TF has a frequent Pfam domain (yes/no)	1/0
DBD	TF has a DNA-binding domain (DBD) (yes/no)	1/0
N_DBD	Number of DBDs (see text)	11/10/00
PPI	TF has a protein-protein interaction (PPI) (yes/no)	1/0
N_PPI	Number of PPIs (see text)	11/10/00
N_PhS	Number of Phosphorylation sites (see text)	11/10/00
PTM	TF has a post-translational modification (PTM) (yes/no)	1/0
Ind_PTM	TF has a specific PTM (yes/no)	An ordered 6-dimensional vector where position i corresponding to PTM i is 1/0
N_ZFD	Number of the zinc finger domains (see text)	11/10/00

#### Encoding of TFClass (TF_Class)

TFClass uses a hierarchical classification, with 10 superclasses at the top level, and a varying number of classes, families, subfamilies etc. below that. We encoded the superclasses (10) and classes (37 in total, ignoring 3 classes with no overlap with our set of TFs) as a 47 bit binary vector, with 1 for the corresponding superclass and class, and 0 elsewhere. This is a reasonable encoding because it will give a Hamming distance of 2 between TFs belonging to different classes within the same superclass, whereas it will be 4 for TFs belonging to different superclasses. Thereby TFs from the same superclass (but different class) will be more similar than TFs from different superclasses. The largest class was class 2.3, the C2H2 zinc finger factors with 475 TFs (the second largest was the homeodomain (3.1) with 199 TFs). To get more balanced subset sizes for the feature vector we extended this into four subclasses (families 2.3.1-4, as family 2.3.5 did not have any overlap with our data).

#### Encoding of frequent Pfam domains (PD)

Transcription factors are typically modular in structure, and will often contain effector domains, one or more DNA-binding domains and other domain types. Type and frequency of domains may reflect the function of a transcription factor.

There were 20 domains with occurrence frequency of more than 20 in the set of TFs mapped to TFClass (see Additional file 1: Table S1). We encoded this as a 20 bit binary vector with each bit corresponding to having a particular Pfam domain or not (1/0).

#### Encoding of DNA binding, PPI, and PTM (DBD, PPI, and PTM)

For encoding of DNA binding, PPI, and PTM as TF properties we encoded each of these properties individually as bits in a binary feature vector, indicating whether it had this property or not (1/0), without taking the number of occurrences into account, i.e. whether it is known to have a PTM or not, and not type or frequency. However, see below for a more detailed encoding.

#### Encoding of individual PTMs (Ind_PTM)

We have previously shown that in particular PTMs may show artificial correlations due to how they are identified [14], and we therefore wanted to test more than one encoding, but with focus on relatively simple encodings that may be more robust to missing data. There were six different types of PTMs annotated in our TF collection; phosphorylation, acetylation, methylation, ubiqutination, sumoylation, and O-GlcNAc. We encoded this for each TF as bits in a binary vector of length six.

#### Encoding of number of DBDs, PPIs, and phosphorylations (N_DBD, N_PPI, and N_PhS)

We extracted the number of DBDs for each TF from our collection. We also used the BioGRID database [24] to extract the number of PPIs for each TF, and added this to our set of properties. From Phosphosite we used the number of sites for each individual modification. We encoded each TF in binary as [1 1] if the number of sites (e.g. for phosphorylations) was higher than the average, [1 0] if it was between one and the average, and [0 0] otherwise. This was done for the number of DBDs (average = 4), the number of PPIs (average = 9), and the number of phosphorylation sites (average = 14). The average for other PTMs was less than 1 and was therefore not considered for extended encoding. This can be seen as a reduced resolution encoding of counts (zero, below average, above average), which is more robust against non-relevant variation than a direct binary encoding of the individual counts.

#### Encoding of the number of frequent zinc finger domains (N_ZFD)

The zinc finger domains are very frequent in TFs, and may therefore require special treatment to get good classification. The Pfam domains zf_C2H2 and zf_H2C2_2 had the highest frequency among zinc fingers. These domains were therefore encoded for the TFs as [1 1] if the TF had more than three of these zinc finger domains, [1 0] if it had between one to three of these domains, and as [0 0] if had none.

### The general classification strategy

#### The classes of regulatory function

In the initial functional classification on chromatin activity there are three main classes; Pioneers, Settlers, and Migrants. An enrichment analysis based on DAVID [25] indicated a functional and structural difference between the Migrants with negative chromatin opening index and the Migrants with positive chromatin opening index (see Results and discussion). For classification we therefore considered them separately, as positive Migrants and negative Migrants.

#### Multiclass classification

The functional classification of additional human TFs is a multiclass classification problem, i.e. classification of patterns into more than two classes. Some classification algorithms are binary algorithms that can be adapted to multiclass classification, whereas other classification algorithms can handle more than two classes by design. There are general strategies for handling the problem of multiclass classification as a binary classification problem [26], and we used a well-known one-vs-rest strategy, which involves training a single classifier per class, with the patterns of that class as positive patterns and all other patterns as negatives. This strategy requires that the base classifier produces a real-valued confidence score for its decision, rather than just a class label, as discrete class labels alone can result in ambiguities, where multiple classes are predicted for a single sample [26]. We used four different cases: Pioneers vs Rest, Settlers vs Rest, positive Migrants vs Rest, and negative Migrants vs Rest.

#### Handling imbalanced data

Any data set that shows an unequal distribution between its classes can be considered as imbalanced. Studies have shown that for several base classifiers, a balanced data set improves the overall classification performance, compared to an imbalanced data set [27, 28]. Using sampling methods on an imbalanced data set, in order to make a balanced one, will therefore normally improve the performance [29].

In this paper we used random under-sampling on training data, without replacement [29]. Specifically, in the Pioneers vs Rest case we randomly split the Migrants and Settlers into 9 subclasses, making 9 different cases, each of them balanced (see Table 2). Random splitting in the Settlers vs Rest case was handled in the same way. For the positive Migrants vs Rest case we randomly split the Rest case into 5 subclasses. For the negative Migrants vs Rest case we randomly split the negative Migrants case into 2 subclasses, and used all Pioneers, Settlers, and positive Migrants as the Rest class. This ensures that each classification problem is balanced, even though the number of cases varies between the classes.

**Table 2:** Summary of criteria for balanced datasets

Case	Specific class	Rest class	Splits	Average size
Pioneers vs Rest	45	47 + 77 + 288	1 + 9	45 + 46
Settlers vs Rest	47	45 + 77 + 288	1 + 9	47 + 46
Positive Migrants vs Rest	77	45 + 47 + 288	1 + 5	77 + 76
Negative Migrants vs Rest	288	45 + 47 + 77	2 + 1	144 + 169

### Classifiers

Several classifiers including Random Forest (RF), Support Vector Classification (SVC) with different kernels (linear, Radial Basis Function (RBF), polynomial), *k* Nearest Neighbors (kNN) and Gaussian Naïve Bayes (GNB) were evaluated, using the one-vs-rest strategy described above. The kNN methods need a specification of the number of neighbors, and the SVC requires parameterization of the complexity constant C and the kernel function. The number of neighbors for kNN was limited to the set {3, 5, 7, 9} [30]. The kNN was performed over all the allowable number of neighbors and the one that had the highest AUC score (see below) was kept. For SVC we considered two kinds of kernel; RBF K(x_i_, x_j_) = exp(−γ(x_i_ − x_j_)^2^) where γ is the width of the RBF function, and polynomial K(x_i_, x_j_) = (x_i_. x_j_)^d^ where d is the degree. A grid search was performed to optimize the parameters of support vector machine (SVM) classifiers. For the RBF kernel, C = {2^− 4^, 2^− 3^, …, 2^3^, 2^4^} and γ = {2^− 4^, 2^− 3^, …, 2^3^, 2^4^} and for polynomial kernel C = {2^− 4^, 2^− 3^, …, 2^3^, 2^4^} and d = {2, 3} .

All machine learning methods were implemented using scikit-learn [31], and all scripts used in the analysis were based on python 2.7 [32].

### Performance measures

We used several common measures to evaluate the performance of the classifiers, including precision (positive prediction value or PPV), recall (sensitivity or SN), F-score, MCC (Matthews’s correlation coefficient), and AUC (area under curve for receiver operating characteristics (ROC)) [33–35]. The true positive (TP) and true negative (TN) cases correspond to TFs correctly predicted as belonging to the specific class or the Rest class, respectively, false positive (FP) denote cases where TFs from the Rest class where predicted as belonging to the specific class, and false negative (FN) denote cases where TFs from the specific class were predicted to the Rest class.

### Cross-validation and comparison of results

The performance of classifiers was tested using five-fold bootstrap cross-validation with ten runs (10 × 5) on all possible rebalanced TF subsets on regulatory function, and for each individual property [36]. In each fold, TFs were randomly sampled as two separate sets: one subset to establish the model (80%, training set) and the rest of the TFs to test the prediction model (20%, test set). The precision, recall, F-score, MCC, and AUC were computed for each run and then averaged over runs for each classifier. It was applied for the four classification cases separately (Pioneers vs Rest, Settlers vs Rest, positive Migrants vs Rest, and negative Migrants vs Rest).

The most frequently used statistical tests to determine significant differences between two machine learning algorithms are the *t*-test and the Wilcoxon test [17]. The *t*-test is a parametric one and requires that the necessary conditions for using it are true, i.e. independence, normality, and heteroscedasticity. This is not the case in the majority of experiments in machine learning [37]. Thus, we investigated the statistical significance of the differences on performance using the nonparametric Wilcoxon test; we kept the result of the AUC measure for each fold and each classifier, and then compared them using Wilcoxon [17].

After identification of the locally best classifier we evaluated the performance on each individual property using the same process as above (bootstrap cross-validation). Again the performance measures were computed for each fold and then averaged on runs for the four classification cases separately.

The final set of properties was selected using a forward best-first search on the list of properties for the four classification cases separately. We executed the runs of each cross-validation and used the average AUC to rank the properties. We started with the property giving the largest AUC, and at each step added the property (among the remaining properties) which results in the best average AUC [38]. We also investigated the statistical significance of the differences of the average AUC in each step after adding a new property, using the paired Wilcoxon tests.

### Analyses using regulatory function

We performed three analyses to illustrate how the predicted regulatory function can be used; enrichment analysis on the subsets of TFs according to regulatory function, co-localization of TFs, and function of TFs involved in a time course experiment.

#### Enrichment analysis

Subsets of TFs were analyzed for enriched properties using DAVID [25] and GOrilla [39], with the set of 1175 TFs with functional classification as background. The set of unclassified TFs was analyzed separately with DAVID, using the full set of 1978 TFs as background.

#### Analysis of TF-TF interactions

We used data on TF-TF interactions from four different sources. Jolma et al. [40] used SELEX with a two-step affinity purification to map TF-TF-DNA interactions, showing that interactions between TFs were predominately mediated by DNA. They identified 315 TF-TF pairs showing cooperative binding (out of 9400 potential pairs), and we used these pairs to identify significant (*p* < 0.05) enrichment (or depletion) for TF-TF interactions within and between the different TF classes, using a 2 × 2 matrix for statistical analysis (i.e., testing for each possible pair of TFs whether the pair represented a specific combination of regulatory functions, and whether there was a known interaction between the pair of TFs). Estimation of *p*-values on the 2 × 2 matrices was done using the fisher.test in R. Further, we used results from the ENCODE Consortium [41], where they used ChIP-seq data to identify pairs of TFs that tend to be co-located to the same genomic regions. For PPIs we used data from Human Protein Reference Database (HPRD) [42] release 9, which includes data on pairwise PPIs for a large number of proteins, including TFs. We also used data from Ravasi et al. [22], where they used a M2H system to systematically screen for PPIs between TFs. This should represent a more coherent dataset than the collection in HPRD, but limited to the experimental conditions used in the M2H system. Data on the number of DNA-binding domains were generated from the list by Bahrami et al. [14]. Data on GC content and IC was generated from matrices downloaded from the Jaspar database [43].

#### Analysis of TFs in a time course experiment

Time course data were made available by the FANTOM Consortium [44, 45], and the assignment of genes to CAGE TSS clusters and edgeR analysis performed by the consortium was used for the project. We analyzed data from an in vitro differentiation time course experiment, generated by Soichi Ogishima and analyzed for expression levels by the FANTOM5 consortium using CAGE (cap analysis of gene expression) [44]. The experiment follows the transition from epithelial cells to mesenchymal cells after induction with TGF-β and TNF-α. The expression levels of individual genes compared to time zero had been analyzed by the FANTOM Consortium using edgeR [46], and we assigned TFs with significantly changed expression level (adjusted *p* < 0.05) to functional classes. Enrichment analysis was done with a Fisher exact test, asking whether a given property was significantly enriched (or depleted) in a given set of TFs, compared to the full set of annotated TFs. The *p*-values were corrected for multiple testing using the Benjamini correction.

## Results and discussion

### Data for training and classification

**Fig. 1:**
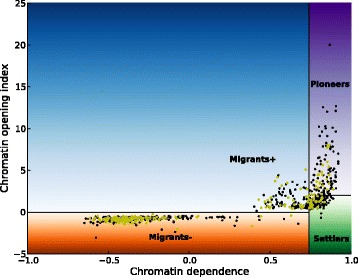
Distribution of TFs based on the classification by Sherwood et al. The classification by Sherwood et al. [10] has been extended to four different functional classes; Pioneers, Settlers, positive Migrants and negative Migrants. The points in *light olive* show the TFs that had intersection with TF classification (TFClass), regulatory function (Sherwood et al.), and our database of TF properties. The figure has been adapted from Sherwood et al

We defined a training set of 457 TFs with known chromatin activity (regulatory function) according to Sherwood et al. [10] and a classification set of 718 TFs, as described in Methods. An initial enrichment analysis of the training subsets using DAVID [25] indicated a functional and structural difference between the Migrants with negative chromatin opening index and the Migrants with positive chromatin opening index (see Additional file 1: Table S2). For classification we therefore considered them separately, and divided our database into four functions: Pioneers (P, 45 TFs), Settlers (S, 47 TFs), positive Migrants (M+, 77 TFs) and negative Migrants (M-, 288 TFs) (see Fig. 1). We encoded TF properties as feature vectors; please see Methods and Table 1 for details and property names.

### Finding the optimal classifier

We first tested the performance of several classifiers using the full set of properties and the one-vs-rest strategy (see Methods) in order to find the classifier with best overall performance on these data. The RF classifier was selected for further analysis as it had the best performance compared to the other classifiers; together with the SVC-RBF classifier it was always ranked as one of the best classifiers in the different cases, but with a higher average AUC score than the SVC-RBF classifier (see Additional file 1: Table S3 for detailed results). Since RF is a multiclass classifier we could in principle also have switched to multiclass classification, rather than the one-vs-rest binarization strategy used so far. However, as shown by Adnan and Islam [47], RF with binarized data actually has a better performance than standard multiclass RF, in particular for cases with more than 3 distinct class values. We therefore decided to keep the one-vs-rest strategy, which also made the subsequent classifications comparable to the initial tests.

### Finding the optimal feature set

**Fig. 2:**
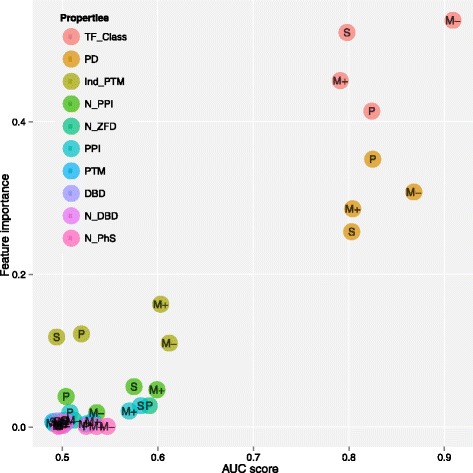
- Important properties for classification. The plot shows RF feature importance and AUC score for each property for the classification of each functional class (Pioneers (*P*), Settlers (*S*), positive Migrants (*M*+) and negative Migrants (*M*-)) versus Rest. This highlights the importance of structural TF classification (TFClass) and Pfam domains (PD) for correct classification of regulatory function. The other properties are DNA binding domain (DBD), number of DBDs (N_DBD), protein-protein interaction (PPI), number of PPIs (N_PPI), post-translational modifications, both in general (PTM) and as individual modifications (Ind_PTM), number of frequent zinc finger domains (N_ZFD) and number of positions for phosphorylation (N_PhS)

We then tested each individual property for classification by estimating the AUC score and the feature importance score, using the RF classifier. The results for the individual properties in the four classification cases are shown in Fig. 2. The data for Fig. 2 are shown in Additional file 1: Table S4, and complete measures of performance on the individual properties are shown in Additional file 1: Table S5. The results showed that the AUC score and the feature importance are highly correlated, and the TF_Class and PD (i.e., Pfam domains) properties gave the highest performance for each of the four binary classification cases. The N_ZFD, PPI and Ind_PTM properties gave the next highest performance for the Pioneers vs Rest, the Settlers vs Rest and Migrants vs Rest cases respectively. The remaining properties gave roughly the same (and lower) performance, often close to random classification (AUC 0.5), even though all the properties were initially selected as potentially relevant for TF function.

**Fig. 3:**
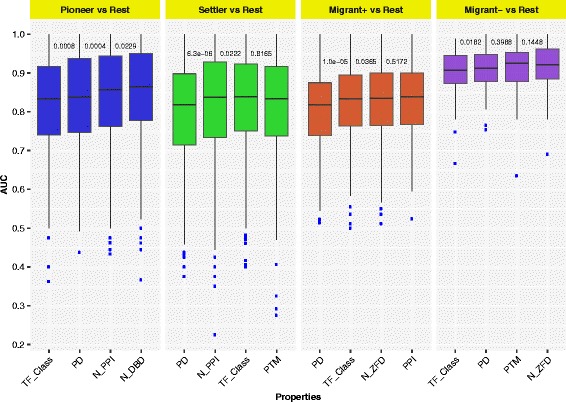
- AUC scores for forward best-first search. The forward best-first algorithm with the RF classifier was used to find a list of properties with good classification performance. The AUC scores while adding properties stepwise (with *p*-values) are shown, at each step adding the property that gave the highest AUC score

Finally we used the forward best-first algorithm with the RF classifier, which should be robust with respect to redundancy between properties. We started with the property that gave the highest AUC score in each case. The process was continued by adding all remaining properties separately to the pervious step, and selecting the property that produced the highest AUC score on a five-fold cross validation over ten runs. Figure 3 shows the general improvement of AUC while stepwise increasing the number of properties, together with the *p*-values (see Additional file 1: Table S6 for details).

The results showed that a list including the TF_Class, PD and N_PPI properties was the best list for the Pioneers vs Rest case; a list including the PD, N_PPI and TF_Class properties was best for the Settlers vs Rest case; a list including the PD, TF_Class, and N_ZFD properties was best for the positive Migrants vs Rest case; and finally for the negative Migrants vs Rest the best list included the TF_Class and PD properties. Interestingly the Ind_PTM property was not selected despite a good ranking based on the feature importance (see Additional file 1: Table S4), indicating that the information may be redundant when other properties are included. The result showed that in particular TF classification and frequent Pfam domains were important features for correct prediction of functional roles. The individual subsets of properties that are listed above were used for the final classification.

Figures 2 and 3 show that the classification is not perfect, according to the AUC score. It is possible that more informative features could have improved the performance. However, it is well documented that TF structure is highly informative on function [12]. It is possible that the non-optimal performance is due to the inherent variation and noise in the biological system, and the fact that the classification of the TFs into subclasses uses cutoff values on continuous variables, which may create some randomness in the classification of borderline cases.

### Classification

The RF classifier was used for the final classification, with the balanced set of 457 TFs with known regulatory function as training data. Classification of the 718 unclassified TFs was predicted over all splits of balanced data as shown in Table 2, and the optimal list of features was used for each case separately. This means that each TF was predicted 9 times for Pioneer vs Rest and Settler vs Rest, 5 times for Positive Migrant vs Rest and 2 times for Negative Migrant vs Rest. Average probability was then computed for each TF for each case separately. The highest probability was used for final classification.

By this strategy we identified 289 TFs as Pioneers, 169 TFs as Settlers, 211 TFs as positive Migrants, and 49 TFs as negative Migrants in the set of 718 unclassified TFs (see Additional file 2: Table S7). The average and margin probability from the adaptive boosting on RF were used to evaluate the quality of the final prediction. A more extensive and in particular experimental validation would require DNase data and binding site models for some of the TFs not included in the original training set. It was not feasible to generate such new data at the current stage, and the validation is therefore limited to the cross-validation performed during training. An overview of the classification result for all the 1175 TFs is given in Table 3.

**Table 3:** Experimental data and classification results according to TFClass

		Total	Experimental					Classified					Average *p*-value	
TFClass	Code	#TFs	#TFs	P	S	M+	M-	#TFs	P	S	M+	M-	All	Margin
Uncharacterized	0.0	6	0	0	0	0	0	6	0	5	1	0	0.682	0.027
NonO domain factors	0.2	2	0	0	0	0	0	2	0	2	0	0	0.679	0.022
Leucine-rich repeat proteins	0.3	1	0	0	0	0	0	1	0	1	0	0	0.679	0.022
NFX1-type putative zf factors	0.4	3	0	0	0	0	0	3	1	2	0	0	0.673	0.016
bZIP	1.1	54	25	2	9	3	11	29	10	9	0	10	0.720	0.150
bHLH	1.2	84	29	1	23	3	2	55	0	55	0	0	0.943	0.539
bHSH	1.3	4	1	1	0	0	0	3	3	0	0	0	0.759	0.128
Nuclear receptors with C4 zfs	2.1	47	47	1	1	45	0	0	0	0	0	0	-	-
Other C4 zfs	2.2	17	4	0	0	1	3	13	0	0	0	13	0.738	0.056
Three-zf Kruppel-related factors	2.3.1	26	5	4	1	0	0	21	21	0	0	0	0.958	0.273
Other factors with 3 adjacent zf	2.3.2	15	0	0	0	0	0	15	12	0	3	0	0.773	0.203
More than 3 adjacent zf	2.3.3	327	12	6	1	4	1	315	201	0	114	0	0.862	0.213
Factors multiple dispersed zf	2.3.4	103	5	1	2	1	1	98	35	58	5	0	0.765	0.086
DM-type intertwined zf factors	2.5	6	0	0	0	0	0	6	0	0	6	0	0.704	0.166
CXXC zf factors	2.6	7	0	0	0	0	0	7	0	0	7	0	0.704	0.112
C2HC zf factors	2.7	8	0	0	0	0	0	8	0	0	8	0	0.704	0.116
C3H zf factors	2.8	2	0	0	0	0	0	2	0	0	2	0	0.704	0.116
C2CH THAP-type zf factors	2.9	1	0	0	0	0	0	1	0	0	1	0	0.704	0.084
Homeo domain factors	3.1	198	198	0	0	3	195	0	0	0	0	0	-	-
Paired box factors	3.2	9	5	0	1	1	3	4	0	1	2	1	0.735	0.181
Fork head	3.3	56	56	2	3	1	50	0	0	0	0	0	-	-
Heat shock factors	3.4	5	0	0	0	0	0	5	0	5	0	0	0.683	0.120
Tryptophan cluster factors	3.5	50	34	23	3	7	1	16	0	0	16	0	0.937	0.264
TEA domain factors	3.6	4	1	0	0	1	0	3	0	0	3	0	0.808	0.152
ARID domain factors	3.7	12	1	0	0	0	1	11	1	10	0	0	0.631	0.012
HMG domain factors	4.1	41	19	0	0	2	17	22	0	0	0	22	0.741	0.338
Het. CCAAT-binding factors	4.2	4	2	2	0	0	0	2	2	0	0	0	0.817	0.357
MADS box factors	5.1	5	2	0	0	0	2	3	0	0	0	3	0.650	0.014
SAND domain factors	5.3	7	3	1	0	2	0	4	0	0	4	0	0.906	0.304
RHR factors	6.1	21	3	0	1	2	0	18	0	0	18	0	0.907	0.125
STAT domain factors	6.2	7	0	0	0	0	0	7	0	0	7	0	0.777	0.076
p53 domain factors	6.3	3	0	0	0	0	0	3	0	0	3	0	0.777	0.062
Runt domain factors	6.4	3	0	0	0	0	0	3	0	0	3	0	0.777	0.062
T-Box factors	6.5	16	1	0	1	0	0	15	0	12	3	0	0.814	0.071
Grainyhead domain factors	6.7	4	0	0	0	0	0	4	0	0	4	0	0.777	0.122
SMAD/NF-1 DBD factors	7.1	8	1	1	0	0	0	7	3	4	0	0	0.761	0.211
GCM domain factors	7.2	2	1	0	1	0	0	1	0	1	0	0	0.844	0.268
TATA-binding proteins	8.1	2	1	0	0	1	0	1	0	0	1	0	0.805	0.171
AT hook factors	8.2	2	1	0	0	0	1	1	0	1	0	0	0.634	0.001
Cold-shock domain factors	9.1	3	0	0	0	0	0	3	0	3	0	0	0.679	0.022
Sum	-	1175	457	45	47	77	288	718	289	169	211	49		

The results are shown for Pioneers (P), Settlers (S), positive Migrants (M+) and negative Migrants (M-)

### Discussion of the classification result

Several interesting observations can be made from the classification results. It is clear that the original dataset by Sherwood et al. is highly biased, in particular with respect to TFClasses 2.3.3 (*Zinc-coordinating DNA-binding domains*/*C2H2 zinc finger factors*/*More than 3 adjacent zinc finger factors*) and 2.3.4 (…/…/*Factors with multiple dispersed zinc fingers*), where the training set contains only 3.7 and 4.9% of the TFs from each class, respectively. Still the classification seems to be quite robust, in particular for 2.3.3 where the average *p*-value is 0.86 and margin is 0.21. This means that the classification presented here provides an important extension of the initial functional classification.

The results show clearly that the regulatory function of a TF to a large extent is determined by how it binds DNA, as the main properties for successful classification are TFClass and Pfam domains. It is also clear that although TFClass is very important for the classification, it is not sufficient by itself, as other properties, in particular Pfam, provide complementary information. This is seen for example in TFClass 2.3.2 (…/…/*Other factors with up to three adjacent zinc fingers*), with 0 cases in the training set, where 15 cases in the full set still could be classified as Pioneers (12) or positive Migrants (3) with reasonable performance (average *p*-value 0.77, margin 0.20). A similar example is TFClass 3.7 (*Helix-turn-helix domains*/*ARID domain factors*). However, the number of TFs in classes with no cases in the training set is very low (76 TFs in total), and in general there is a good distribution of cases in the training set. This is reflected in the classification result as several cases of high average *p*-values. An example is TFClass 3.5 (…/*Tryptophan cluster factors*), with an average *p*-value of 0.94 and a margin of 0.26. This indicates a quite reliable classification.

However, the classification is clearly not perfect, with an AUC score in the range of 0.82–0.92. It is difficult to say whether the main reason is imperfect training set, key features lacking in the property set, or non-optimal classifiers. However, it is clear that the best performance is seen for the negative Migrants, which also represent the most well-defined subset (see Fig. 1). This may indicate that the initial classification by Sheerwood et al. can be improved. However, this aspect has not been investigated further.

### Analyses using regulatory function

We then used the full set of TFs that could be classified as Pioneers, Settlers or Migrants for three types of data analysis; enrichment analysis on regulatory function, analysis of properties associated with TF-TF and TF-DNA interactions for the individual classes of regulatory function, and finally a time course experiments where several TFs show changes in expression level.

#### General properties associated with regulatory function

Subsets according to regulatory function were analyzed for enriched properties using DAVID [25] and GOrilla [39], using the set of 1175 TFs as background. The set of unclassified TFs was analyzed separately with DAVID to test for any potential bias, using the full set of 1978 TFs as background.

For DAVID we looked in particular into the Functional Annotation Clustering output, which groups together associated terms from different annotation sources into functional clusters, based on enrichment (see Additional file 3: Table S8). For the unclassified TFs the most enriched terms where “chromatin modification” and similar terms, followed by for example “protein complex assembly”, “transcription initiation”, “RNA processing” and “DNA repair”. This seems to indicate that the unclassified TFs were not enriched for regulatory TFs, but rather general TFs and proteins associated with TFs and other regulatory functions. There was no strong indication of any problematic bias associated with the unclassified TFs.

For Pioneers all the most enriched clusters were related to DNA-binding domains and their properties, such as “zinc fingers”, “metal binding”, KRAB, BTB, ETS etc. More functional terms like “cell cycle” or “cancer” were found only at quite low enrichment. This was confirmed by GOrilla (see Additional file 1: Table S9), where the most enriched term was “metal ion binding”, and no terms related to function were enriched. For Settlers the picture was similar, with highest enrichment for domains like HLH, PAS, TBOX and bZIP, which also was confirmed by GOrilla, where the most enriched term was “protein dimerization activity”. However, in DAVID also more functional terms like “tissue morphogenesis” and “response to stimulus” were clearly enriched. This trend was even clearer for the positive Migrants. Although terms like “zinc finger” and “metal ion binding” were strongly enriched, so were also terms like “ligand binding”, “hormone receptor”, “lipid binding” and “signaling pathway”, and for GOrilla the most enriched term was “receptor activity”. A similar picture was seen in negative Migrants. Here “fork head” and POV was strongly enriched, but for example “cell motility”, “cell migration”, “cell morphogenesis” and “axonogenesis” showed a similar enrichment. For GOrilla “chromatin binding” was the most enriched term.

This seems to indicate functional roles of these classes of TFs that are consistent with what previously has been assumed, where Pioneers may have a very general role in initiating gene regulation, independent of specific biological processes. Then Settlers may be somewhat closer to biological process, whereas positive and negative Migrants are even more closely linked to specific processes, in particular signaling and differentiation, respectively.

The fact that similar DBDs tend to be associated with the same functional class may be consistent with a hypothesis suggesting that TFs from different functional classes bind to cis-regulatory regions in a hierarchical process, rather than by competing for the same binding site(s) [10].

#### Analysis of TF-TF interactions

**Fig. 4:**
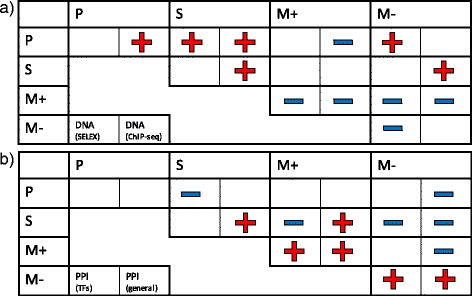
– Enrichment and depletion in TF-TF interactions. The figure illustrates cases of enrichment (*red plus*) and depletion (*blue minus*) relative to random expectation for TF-TF interactions between classes of regulatory function, based on data related to **a** DNA binding and **b** PPI. For each pair of regulatory functions (Pioneers (*P*), Settlers (*S*), positive Migrants (*M*+) and negative Migrants (*M*-)) the enrichment or depletion is indicated for each of the data sources, 2× DNA binding in **a**) and 2× PPI in **b**). The strongest tendency is that interactions involving in particular Pioneers tend to be enriched in DNA-based interactions, whereas interactions involving in particular positive Migrants tend to be depleted in DNA-based interactions and enriched in PPIs

Binding of several TFs to a regulatory region is an important process for gene activation. Interactions between TFs can take place either because the TFs tend to bind to neighboring binding sites in DNA, or because they tend to interact through protein-protein interactions, or both, and it seems likely that the relative importance of this may differ with regulatory function. We therefore used data from two different sources (SELEX and ChIP-seq) on TF-TF interaction through co-localization to DNA, and on TF-TF interaction through PPI, also from two different sources (TF-specific and general, see Methods for details). These data were tested for enrichment or depletion of the functional subclasses. The results are illustrated in Fig. 4.

As expected, the results are somewhat noisy, as in particular PPI data are known to be affected by large fractions of false positives, e.g. due to non-specific binding. However, the results are fairly consistent within the two main interaction types (TF co-localization and PPI), and the overall trend is also quite clear; TF co-localization and PPI are to some extent mutually exclusive features. Whereas Pioneers and Settlers tend to be enriched for TF-TF-DNA interactions and depleted for PPI, the Migrants (and in particular positive Migrants) are enriched for PPI and depleted for TF-TF-DNA. This is consistent with what we see at a more detailed level. In the data on TF-TF-DNA interactions we see for example a clear depletion of interactions between Pioneers like ETV2 and ETV5 and positive Migrants (log ratios of observed vs expected are −1.12 and −1.23, respectively), whereas the Settler MAX is enriched for interaction with other Settlers (log ratio 1.00). The positive Migrant TEAD4, which is known to bind non-cooperatively [48], is depleted for interaction with other positive Migrants (log ratio −1.08). In the PPI data we see that positive Migrants like RXRB and RXRG are enriched for interaction with other positive migrants (log ratio 1.41 and 1.25, respectively), and negative Migrants like ALX4 and POU2F1 are enriched for interaction with other negative Migrants (log ratio 1.17 and 1.25, respectively). However, there seems to be a lack of frequent interactions between individual Pioneer TFs and other TFs, in particular for PPIs, probably indicating that most Pioneers may enable transcription through other mechanisms, for example by initiating chromatin remodeling [49].

In order to explain our observations we looked at three properties that may influence TF-binding to DNA; the number of DNA-binding domains in the TF, the GC-content of the binding site, and the Information Content (IC) of the binding site motif. In all three cases we split the set of TFs into two, based on a suitable cutoff value for the relevant property, and tested each functional class of TFs for enrichment or depletion. The results are shown in Table 4.

**Table 4:** Enriched or depleted features related to TF-TF-DNA interactions

	Obs/Exp	*P*	Average
TFs with #DBDs > 1			#DBDs
Pioneers	278/152	<2.2e-16	7.05
*Settlers*	*61/98*	*1.1e-08*	*2.87*
Migrants+	145/131	0.07	4.80
*Migrants-*	*51/153*	*<2.2e-16*	*1.23*
TFs with GC > 40%			%GC
Pioneers	45/16	1.4e-11	60.7
Settlers	47/17	6.9e-11	58.8
Migrants+	71/28	5.3e-11	52.6
*Migrants-*	*9/108*	*1.8e-10*	*23.7*
TFs with IC > 9.0			IC
Pioneers	38/23	3.2e-06	10.4
Settlers	29/24	0.21	10.3
*Migrants+*	*20/40*	*3.4e-07*	*7.7*
Migrants-	153/151	0.77	9.3

Significantly *depleted* features are highlighted in *italics*

For the number of TFs with more than one DBD, we saw a very significant enrichment in Pioneers, and a clear depletion in Settlers and negative Migrants, which means that Pioneers often will have a very strong and specific binding, compared to the other TFs. With respect to GC content, the negative Migrants were strongly depleted for binding sites with high GC content, whereas the other TFs were enriched. The general enrichment is consistent with previous results [50] showing that many TF binding sites have a high GC-content, and that this also favors binding and positioning of nucleosomes. The high GC-content of Pioneer binding sites is consistent with this, as the Pioneers are more likely to be involved in chromatin opening and repositioning of nucleosomes. This strengthens the significance of the depletion seen in negative Migrants, where the low GC-content in binding sites may be consistent with their preference for open chromatin without stably bound nucleosomes. Finally, the analysis of information content showed that Pioneers are enriched for high IC, whereas positive Migrants are depleted. The result for Pioneers is consistent with their role in initiating chromatin opening at specific genomic positions, requiring a more specific motif than other TFs, in particular Migrants.

The results suggest that the chromatin opening index of Sherwood et al. is associated with the number of DBDs and possibly IC of the motif, whereas the chromatin dependence is associated with GC content of the binding sites. The enrichment for multiple DBDs in Pioneers and the corresponding depletion in Settlers and in particular negative migrants coincides mainly with the axis for chromatin opening index in Fig. 1. The depletion for high GC content in negative Migrants and the corresponding enrichment in Pioneers, Settlers and positive Migrants coincides mainly with the axis for chromatin dependence. The pattern for IC is less clear, given the enrichment for high IC in Pioneers and corresponding depletion in positive Migrants.

The results from this analysis can be summarized as a scenario for how TFs may cooperate to enable gene expression. As a first step activating Pioneers can bind strongly and with high specificity (due to multiple DBDs with high IC) to nucleosome-bound regions (due to high GC) to initiate chromatin remodeling, leading to at least partly open chromatin. This is followed by Settlers, which are quite similar to Pioneers, but with fewer DBDs. They are therefore less likely to compete directly with nucleosomes for binding, at least at an individual level, but can stabilize open chromatin through cooperative binding. This process may be supported by the positive Migrants, which are somewhat similar to Settlers, although they have on average more DBDs but with lower IC, and they are more likely to interact through PPIs. This may give more general (less specific) binding in regions of open chromatin, which may contribute to stabilizing these regions in an open state. Finally, the negative Migrants have few DBDs and low GC content, which means that they can bind to more AT-rich regions as found in for example linkers [51], and their binding may be stabilized through PPIs. This scenario provides an interesting basis for further testing and verification.

#### Analysis of TFs in a time course experiment

We analyzed data from an in vitro differentiation time course experiment following the transition from epithelial cells to mesenchymal cells after induction with TGF-β and TNF-α. TFs with significantly changed expression level (*p* < 0.05) were assigned to classes of regulatory function. The number of TFs showing significant changes in expression level at each time point are shown in Table S10 (see Additional file 1). All groups of TFs followed a similar time course where they were rapidly upregulated, followed by a relaxation leading to what seems to be a net downregulation in number of expressed genes (see Additional file 1: Figure S1). This may reflect a rapid activation of new genes in the regulatory network, with no clear distinction between TFs with different regulatory function. Since Pioneers, Settlers and Migrants are all needed for this, it is not surprising that they seem to be regulated in parallel.

We then used our list of TF properties [14], including e.g. Pfam domains and post-translational modifications, to check whether there were significant enrichment of specific properties associated with TFs in this experiment, using *p*-values after Benjamini correction for multiple testing (see Table 5). It should be noted that although we are partly using the same properties as for the classification, we are here testing for enrichment in the specific subset of TFs that show significant changes in expression levels in this particular experiment, rather than across all TFs.

**Table 5:** Enriched or depleted features in significantly regulated TFs

Class	Up			Down		
	Term	Obs/Exp	P(Benj)	Term	Obs/Exp	P(Benj)
Pioneers	Ets	9/0	2.2e-07			
	zf-H2C2_2	25/9	1.2e-05	zf-H2C2_2	69/18	1.0e-08
				KRAB	36/10	1.1e-08
				zf-H2C2_4	14/3	9.2e-05
Settlers	HLH	20/1	2.5e-09	HLH	18/2	3.9e-09
				Ubiquitination	33/18	4.7e-04
				Sumoylation	11/5	4.6e-02
				*PPI*	*5/11*	*4.9e-02*
Migrants+	PPI	20/11	6.1e-03			
				Hormone_recep	19/1	6.6e-09
				zf-C2H2	33/8	7.3e-09
				zf-C4	19/1	9.7e-09
Migrants-				HMG_box	11/1	1.1e-05
				*zf-H2C2_2*	*1/14*	*3.6e-04*
				Homeobox	17/5	9.3e-04
				Methylation	16/8	4.2e-02
All	Ubiquitination	74/49	5.4e-05	Ubiquitination	112/90	8.4e-03
	bZIP_1	12/2	1.8e-04			
	HLH	20/6	2.7e-04			
	Ets	10/2	1.1e-03			
	Sumoylation	27/15	5.1e-03			
	PPI	50/37	1.8e-02			
	Phosphorylation	144/136	4.9e-02	Phosphorylation	269/252	7.7e-04
				zf-H2C2_2	113/63	5.7e-08
				zf-C2H2	56/29	6.0e-05
				zf-C4	20/6	4.8e-05
				Hormone_recep	20/6	8.4e-05
				KRAB	58/34	8.9e-04
				zf-C2H2_4	24/10	2.5e-03

Only features with at least 9 occurrences (as observed for enrichments or expected for depletions) are listed. Depleted features are highlighted in *italics*. The All category shows enrichment analysis of all significantly up- or down-regulated genes, independent of functional classification

This analysis revealed several interesting features, in particular for Pioneers where there seems to be a shift in the regulatory program. The Pfam Ets domain is enriched in up-regulated Pioneers, whereas KRAB is enriched in down-regulated. The KRAB domain is associated with transcriptional repression, as it interacts with a corepressor protein (KAP-1) which recruits histone deacetylases and chromatin remodeling complexes to chromatin [52], maintaining a repressed status. This indicates that transcriptional repression is actively released in the differentiation process, enabling activation of new genes. This is similar to what has been observed for KRAB-containing TFs in several processes involving metabolic changes or differentiation [52]. The Ets domain, which is enriched in up-regulated Pioneers, can act both as an activator and a repressor [53], and is known to be involved in differentiation of e.g. T-cells [54]. However, another point here could be that many Ets-containing TFs are down-stream targets of signal transduction cascades [53], indicating an up-regulation of responses to signaling.

As already indicated, most of the observed changes are linked to down-regulation. For example, down-regulated Settlers are enriched in ubiquitination, a post-translational modification that may target proteins for degradation, possibly leading to a more rapid and efficient down-regulation of TFs than by changing the transcript level alone. For up-regulated positive Migrants there is enrichment for PPIs, supporting the observation above regarding PPIs and positive Migrants. This is possibly linked to stabilization of protein complexes involved in regulation of transcription.

A couple of Pfam domains (zf-H2C2_2 and HLH) are enriched in both up-regulated and down-regulated sets. The zinc-finger domain zf-H2C2, which is often involved in sequence-specific targeting of other domains, including KRAB [52], is strongly enriched in Pioneers, indicating site-specific changes in gene regulation. This is not seen for the Settlers, although the HLH domain may play a similar role here. For the positive Migrants it is seen only for the down-regulated TFs, whereas the negative Migrants actually are strongly depleted for zf-H2C2 domains. This illustrates a clear difference between the sequence-specific targeting of Pioneers, compared to other TFs where additional interactions may be important.

We also did the same analysis over all TFs, independent of functional classification (Table 5). This identified most of the same terms as enriched, but at lower significance, and not the two depletions. Also, the analysis using classes of regulatory function clearly linked several changes in enrichment of properties to specific classes, such as Ets to Pioneers and PPI to positive Migrants. This is additional information that may help in interpretation of results, and underlines the added benefit of including data on regulatory function.

The results described above seem to support a general picture of these TFs that is consistent with their assumed roles. The Pioneers are rapidly regulated to modify the transcriptional program, mainly by removing repressing TFs and up-regulating activating TFs that bind in a sequence-specific manner. This process is supported by the regulation of Settlers, many of which are rapidly degraded and removed, possibly to close up the regulatory regions that are being de-activated. The up-regulated Migrants are enriched for protein-protein interactions, which may support the formation of clusters of TFs in open regulatory regions.

## Conclusions

Data on properties of transcription factors has been used as input for supervised machine learning in order to expand an experimental classification of transcription factors into functional classes associated with chromatin opening, as Pioneers, Settlers, positive and negative Migrants. The expanded classification is a useful resource for analyzing other data. Here is has been used to analyze transcription factor interaction data and data from a time course experiment. The results are consistent with the expected roles of the functional TF classes, in particular the role of Pioneers in initiating changes in gene regulation.

## Additional files

Additional file 1: Table S1. Frequent Pfam domains in TF data; **Table S2** - DAVID analysis on experimentally identified subclasses of regulatory function; **Table S3** - AUC score for classifiers on each of the four cases; **Table S4** - AUC score and feature importance by RF classifier on individual properties; **Table S5** - Performance measures on individual properties using the RF classifier; **Table S6** - Significance of change in AUC score for stepwise addition of properties; **Table S9** - GOrilla analysis on final classification data; **Table S10** - Number of significantly up- or down-regulated TFs at each time point; **Figure S1** - Log ratio of number of up-regulated versus down-regulated TFs. (PDF 232 kb)
Additional file 2: Table S7. Full list of transcription factors, with properties and their classification. (XLS 945 kb)
Additional file 3: Table S8. Functional Annotation Clustering output from DAVID enrichment analysis. (XLS 1695 kb)

## Abbreviations

AUC: Area under curve (normally receiver operating characteristic curve)
CAGE: Cap analysis of gene expression
ChIP-seq: Chromatin immunoprecipitation and sequencing
DBD: DNA-binding domain
FPR: False positive rate
GNB: Gaussian naïve Bayes
HTH: Helix-turn-helix
IC: Information content
kNN: k-nearest neighbor
LinearSVC: Linear Support Vector Classification
MCC: Matthews correlation coefficient
PFM: Position frequency matrix
PIQ: Protein interaction quantitation
PPI: Protein-Protein Interaction
PPV: Positive prediction value
PTM: Post-translational modifications
RBF: Radial basis function
RF: Random forest
ROC: Receiver operating characteristic
SN: Sensitivity
SVM: Support vector machine
TF: Transcription factor
TFBS: Transcription factor binding site
TN: True negative
TP: True positive
TPR: True positives rate
TRANSFAC: TRANScription FACtor database
