# Supplementary material for: Feature-based classification of human transcription factors into hypothetical sub-classes related to regulatory function
Source: BMC Bioinformatics. 2016 Nov 14;17:459. doi: 10.1186/s12859-016-1349-2 (PMC5109715; doi:10.1186/s12859-016-1349-2)
Supplement: Additional file 1: Table S1. — Frequent Pfam domains in TF data; Table S2 - DAVID analysis on experimentally identified subclasses of regulatory function; Table S3 - AUC score for classifiers on each of the four cases; Table S4 - AUC score and feature importance by RF classifier on individual properties; Table S5 - Performance measures on individual properties using the RF classifier; Table S6 - Significance of change in AUC score for stepwise addition of properties; Table S9 - GOrilla analysis on final classification data; Table S10 - Number of significantly up- or down-regulated TFs at each time point; Figure S1 - Log ratio of number of up-regulated versus down-regulated TFs. (PDF 232 kb) [file 12859_2016_1349_MOESM1_ESM.pdf]

Additional File 1

R. Ehsani, S. Bahrami and F. Drabløs: Functional classification of human transcription factors based on structural properties

Table S1 - Frequent Pfam domains in TF data

Table S2 - DAVID analysis on experimentally identified subclasses of regulatory function

Table S3 - AUC score for classifiers on each of the four cases

Table S4 - AUC score and feature importance by RF classifier on individual properties

Table S5 - Performance measures on individual properties using the RF classifier

Table S6 - Significance of change in AUC score for stepwise addition of properties

Table S7 - Classification of individual TFs
(see Additional File 2)

Table S8 - DAVID analysis on final classification data
(see Additional File 3)

Table S9 - GOrilla analysis on final classification data

Table S10 - Number of significantly up- or down-regulated TFs at each time point

Figure S1 - Log ratio of number of up-regulated versus down-regulated TFs

Table S1 - Frequent Pfam domains in TF data

Pfam ID	Name	Frequency
PF13465	Zf-H2C2_2	3466
PF00096	Zf-C2H2	347
PF01352	KRAB	237
PF00046	Homeobox	195
PF13894	Zf-C2H2_4	127
PF13912	Zf-C2H2_6	88
PF00010	HLH	74
PF00104	Hormone_recep	47
PF00105	Zf-C4	45
PF02023	SCAN	44
PF00250	Fork_head	42
PF00505	HMG_box	42
PF00651	BTB	39
PF00170	Bzip_1	30
PF13909	Zf-H2C2_5	29
PF00023	Ank	26
PF00178	Ets	25
PF00412	LIM	22
PF00628	PHD	22
PF01530	Zf-C2HC	21

Table S2 - DAVID analysis on experimentally identified subclasses of regulatory function

Subclass	Category	Term	Count	%	P-value	Benjamini
Pioneer	INTERORP	Zinc finger, C2H2-type/integrase, DNA-binding	10	22.7	1.8E-4	2.0E-3
	SP_PIR_KEYWORDS	Activator	27	61.4	3.9E-4	1.7E-2
	GOTERM_MF_FAT	Transcription cofactor activity	14	31.8	1.6E-3	7.6E-2
	PFAM	Ets	22	50.0	2.6E-21	1.5E-19
	PFAM	SAM_PNT	7	15.9	3.6E-5	6.9E-4
	PFAM	zf-C2H2	11	25.0	1.7E-3	2.5E-2
Settler	UP_SEQ_FEATURE	DNA-binding region: Basic motif	30	65.2	1.8E-20	3.0E-18
	SP_PIR_KEYWORDS	Transmembrane	8	17.4	1.9E-6	5.9E-5
	SP_PIR_KEYWORDS	Activator	29	63.0	1.1E-4	1.7E-3
	SP_PIR_KEYWORDS	Unfolded protein response	5	10.9	4.0E-4	5.0E-3
	PFAM	HLH	22	47.8	2.3E-18	1.2E-16
	PFAM	bZIP_1	7	15.2	9.6E-5	1.7E-3
Positive Migrant	SP_PIR_KEYWORDS	Receptor	45	57.9	4.5E-36	3.3E-34
	GOTERM_MF_FAT	Steroid hormone receptor activity	44	57.9	4.9E-36	4.6E-34
	GOTERM_MF_FAT	Transcription activator activity	34	44.7	2.8E-5	3.7E-4
	SP_PIR_KEYWORDS	Phosphoprotein	45	59.2	3.7E-5	3.8E-4
	GOTERM_MF_FAT	Transcription coactivator activity	15	19.7	2.2E-4	2.6E-3
	GOTERM_MF_FAT	Transcription factor binding	29	38.2	2.4E-4	2.5E-3
	PFAM	Hormone_recep	43	56.6	6.9E-35	2.6E-33
	PFAM	zf-C4	42	55.3	7.7E-34	1.9E-32
Negative Migrant	GOTERM_MF_FAT	Sequence-specific DNA binding	260	90.3	1.3E-10	8.6E-9
	COG_ONTOLOGY	Transcription	16	5.6	1.0E-6	1.0E-6
	GOTERM_MF_FAT	Transcription factor activity	279	96.9	6.7E-5	2.3E-3
	PFAM	Homeobox	192	66.7	2.2E-48	2.6E-46
	PFAM	Fork_head	39	13.5	1.7E-7	6.7E-6

Table S3 - AUC score for classifiers on each of the four cases

Methods	Pioneers vs Rest	Settlers vs Rest	Migrants+ vs Rest	Migrants- vs Rest
RF	*0.830*	*0.801*	*0.804*	*0.903*
SVC_Linear	0.818	0.793	0.786	0.890
SVC_RBF	0.824	0.798	0.800	0.896
SVC_Poly	0.503	0.506	0.506	0.503
kNN	*0.825*	0.788	*0.806*	0.888
DT	0.808	0.771	0.791	*0.901*
GNB	0.815	*0.800*	0.774	0.856

The two best scores for each case are shown in *italics*. The methods are Random Forest (RF), Support Vector Classifier with Linear (SVC_Linear), Radial Basis Function (SVC_RBF) and Polynomial (SVC_Poly) kernels, k Nearest Neighbours (kNN), Decision Tree (DT), and Gaussian Naïve Bayes (GNB).

Table S4 - AUC score and feature importance by RF classifier on individual properties.

Properties	Pioneers vs Rest		Settlers vs Rest		Migrants+ vs Rest		Migrants- vs Rest	
	**AUC score**	**Feature importance**	**AUC score**	**Feature importance**	**AUC score**	**Feature importance**	**AUC score**	**Feature importance**
TF_Class	*0.824*	*0.414*	*0.798*	*0.517*	*0.791*	*0.454*	*0.909*	*0.533*
PD	*0.825*	*0.351*	*0.803*	*0.256*	*0.804*	*0.286*	*0.868*	*0.308*
Ind_PTM	0.520	*0.122*	0.494	*0.118*	*0.603*	*0.161*	*0.612*	*0.110*
N_PPI	0.504	0.040	0.575	0.053	0.599	0.049	0.536	0.019
N_ZFD	*0.591*	0.028	0.502	0.009	0.491	0.005	0.512	0.009
PPI	0.508	0.019	*0.582*	0.028	0.570	0.021	0.506	0.008
PTM	0.498	0.006	0.490	0.007	0.532	0.008	0.525	0.004
DBD	0.495	0.008	0.502	0.004	0.499	0.005	0.498	0.002
N_DBD	0.504	0.007	0.497	0.002	0.499	0.005	0.500	0.002
N_PhS	0.525	0.000	0.496	0.000	0.536	0.000	0.547	0.000

The three best scores for each case are shown in *italics*. The individual properties are explained in Table 1.

Table S5 - Performance measures on individual properties using the RF classifier.

a) Pioneer vs rest

Property	PPV	SN	F-Score	TP-ave	FP-ave	TN-ave	FN-ave	MCC	AUC
TFClass	0.844	0.804	0.801	7.19	1.44	7.56	1.80	0.651	0.824
PD	0.854	0.800	0.799	7.16	1.44	7.57	1.83	0.658	0.825
N_ZFD	0.692	0.349	0.379	3.00	1.65	7.35	5.99	0.232	0.591
DBD	0.195	0.420	0.261	3.48	4.18	4.83	5.51	-0.018	0.495
N-DBD	0.298	0.360	0.247	2.94	3.46	5.54	6.05	0.018	0.504
PPI	0.394	0.406	0.347	3.42	3.70	5.31	5.58	0.019	0.508
N-PPI	0.420	0.393	0.340	3.34	3.62	5.38	5.65	0.009	0.504
N_PhS	0.502	0.440	0.411	3.84	3.64	5.36	5.15	0.059	0.525
PTM	0.257	0.467	0.308	3.90	4.52	4.49	5.50	-0.008	0.498
Ind-PTM	0.518	0.433	0.428	3.80	3.61	5.39	5.19	0.040	0.520

b) Settler vs rest

Property	PPV	SN	F-Score	TP-ave	FP-ave	TN-ave	FN-ave	MCC	AUC
TFClass	0.825	0.784	0.778	7.16	1.70	7.16	1.99	0.604	0.798
PD	0.811	0.833	0.794	7.66	2.00	6.58	1.49	0.624	0.803
N_ZFD	0.332	0.458	0.323	4.04	4.18	4.67	5.10	0.006	0.502
DBD	0.327	0.450	0.309	3.94	4.14	4.71	5.21	0.006	0.502
N-DBD	0.255	0.464	0.305	4.07	4.34	4.51	5.08	-0.010	0.497
PPI	0.467	0.770	0.568	6.94	5.43	3.42	2.21	0.204	0.582
N-PPI	0.467	0.776	0.570	6.98	5.63	3.22	2.16	0.186	0.575
N_PhS	0.463	0.482	0.419	4.34	4.42	4.43	4.81	-0.007	0.496
PTM	0.329	0.513	0.354	4.55	4.68	3.99	4.60	-0.028	0.490
Ind-PTM	0.505	0.473	0.451	4.31	4.37	4.48	4.84	-0.010	0.494

c) Positive Migrant vs rest

Property	PPV	SN	F-Score	TP-ave	FP-ave	TN-ave	FN-ave	MCC	AUC
TFClass	0.819	0.757	0.772	11.51	2.62	12.14	3.74	0.558	0.791
PD	0.816	0.810	0.792	12.30	3.03	11.72	2.94	0.618	0.804
N_ZFD	0.370	0.504	0.351	7.44	7.98	6.77	7.81	-0.027	0.491
DBD	0.336	0.525	0.353	7.74	8.05	6.70	7.51	-0.003	0.499
N-DBD	0.332	0.507	0.341	7.46	7.79	6.96	7.78	-0.003	0.499
PPI	0.588	0.475	0.485	7.12	5.06	9.69	8.13	0.148	0.570
N-PPI	0.678	0.458	0.498	6.91	3.92	10.38	8.34	0.222	0.599
N_PhS	0.514	0.616	0.499	9.20	8.20	6.55	6.05	0.103	0.536
PTM	0.365	0.683	0.469	10.20	9.34	5.41	5.05	0.101	0.532
Ind-PTM	0.637	0.524	0.544	7.94	4.75	10.00	7.30	0.213	0.603

d) Negative Migrant vs rest

Property	PPV	SN	F-Score	TP-ave	FP-ave	TN-ave	FN-ave	MCC	AUC
TFClass	0.906	0.903	0.902	26.71	2.79	30.13	2.87	0.819	0.909
PD	0.934	0.795	0.852	23.45	1.93	30.99	6.13	0.753	0.868
N_ZFD	0.142	0.304	0.192	8.30	9.87	23.05	21.28	0.045	0.512
DBD	0.051	0.118	0.071	3.12	4.38	28.54	26.46	-0.006	0.498
N-DBD	0.082	0.151	0.094	4.06	5.37	27.55	25.52	0.000	0.500
PPI	0.152	0.233	0.176	6.42	7.69	25.23	23.16	0.011	0.506
N-PPI	0.603	0.281	0.266	7.83	7.23	25.69	21.75	0.117	0.536
N_PhS	0.577	0.334	0.346	9.73	8.02	24.90	19.85	0.127	0.547
PTM	0.584	0.125	0.182	3.64	2.52	30.40	25.94	0.083	0.525
Ind-PTM	0.576	0.650	0.596	19.03	14.13	18.79	10.55	0.227	0.612

Table S6 - Significance of change in AUC score for stepwise addition of properties.

Pioneers vs Rest										
Properties	TF_Class	PD	N_PPI	N_DBD	N_ZFD	DBD	PPI	PTM	Ind_PTM	N_PhS
AUC-Score	0.8223	0.8315	0.8404	0.8459	0.8444	0.8456	0.8430	0.8451	0.8373	0.8330
P-value		0.0008	0.0004	0.0229	0.4139	0.8206	0.1055	0.1378	0.0122	0.0395
**Settlers vs Rest**										
Properties	PD	N_PPI	TF_Class	PTM	N_DBD	N_ZFD	N_PhS	PPI	DBD	Ind_PTM
AUC-Score	0.8002	0.8180	0.8224	0.8211	0.8220	0.8251	0.8230	0.8168	0.8129	0.8024
P-value		6.3e-06	0.0222	0.8165	0.4149	0.3442	0.3659	0.0197	0.0258	0.0040
**Positive Migrants vs Rest**										
Properties	PD	TF_Class	N_ZFD	PPI	DBD	N_DBD	PTM	N_PPI	Ind_PTM	N_PhS
AUC-Score	0.8053	0.8238	0.8290	0.8269	0.8280	0.8241	0.8133	0.8059	0.7993	0.8039
P-value		1.0e-05	0.0365	0.5172	0.6968	0.0438	0.0003	0.0232	0.0586	0.7215
**Negative Migrants vs Rest**										
Properties	TF_Class	PD	PTM	N_ZFD	N_DBD	DBD	PPI	N_PhS	N_PPI	Ind_PTM
AUC-Score	0.9063	0.9131	0.9157	0.9172	0.9176	0.9139	0.9101	0.9117	0.9071	0.8946
P-value		0.0182	0.3988	0.1448	0.8565	0.0351	0.2052	0.0940	0.0475	0.0004

Table S7 - Classification of individual TFs
(see Additional File 2)

The classification is shown as Chromatin Opening Type. The experimentally determined classifications used for training the classifiers are shown in lowercase (e.g. Pioneer), whereas the classifications described here are shown in UPPERCASE (e.g. PIONEER).

Table S8 - DAVID analysis on final classification data.
(see Additional File 3)

The table contains the full output from Functional Annotation Clustering in DAVID for Pioneers, Settlers, positive Migrants and negative Migrants, using the set of 1175 TFs as background. It also contains the output for the Unclassified TFs, using the full set of 1978 TFs as background.

Table S9 - GOrilla analysis on final classification data.

GO term	Description	P-value	q-value	Enrichment
**Pioneers**				
GO:0043169	cation binding	2.11E-50	1.05E-47	1.63
GO:0046872	metal ion binding	2.11E-50	5.25E-48	1.63
GO:0043167	ion binding	5.01E-49	8.32E-47	1.61
GO:1901363	heterocyclic compound binding	2.12E-04	2.64E-02	1.02
GO:0003676	nucleic acid binding	2.12E-04	2.11E-02	1.02
GO:0097159	organic cyclic compound binding	2.12E-04	1.76E-02	1.02
**Settlers**				
GO:0046983	protein dimerization activity	1.04E-19	5.19E-17	2.33
GO:0070888	E-box binding	1.93E-13	4.80E-11	4.46
GO:0035497	cAMP response element binding	3.51E-07	5.83E-05	4.93
GO:0008134	transcription factor binding	2.19E-05	2.73E-03	1.62
GO:0005515	protein binding	5.29E-05	5.27E-03	1.19
GO:0000988	transcription factor activity, protein binding	1.20E-04	9.99E-03	1.53
GO:0000989	transcription factor activity, transcription factor binding	3.12E-04	2.22E-02	1.50
GO:0043425	bHLH transcription factor binding	7.27E-04	4.53E-02	2.86
**positive Migrants**				
GO:0004872	receptor activity	7.24E-25	3.61E-22	3.69
GO:0038023	signaling receptor activity	7.24E-25	1.8E-22	3.69
GO:0003707	steroid hormone receptor activity	1.62E-24	2.68E-22	3.75
GO:0004871	signal transducer activity	5.75E-24	7.16E-22	3.27
GO:0060089	molecular transducer activity	5.75E-24	5.73E-22	3.27
GO:0098531	transcription factor activity, direct ligand regulated sequence-specific DNA binding	2.13E-21	1.52E-19	3.71
GO:0046914	transition metal ion binding	1.09E-18	6.77E-17	2.27
GO:0008270	zinc ion binding	8.41E-18	4.65E-16	2.25
GO:0043167	ion binding	6.15E-17	3.06E-15	1.39
GO:0043169	cation binding	4.17E-15	1.89E-13	1.37
GO:0046872	metal ion binding	4.17E-15	1.73E-13	1.37
GO:0008289	lipid binding	1.41E-09	5.39E-08	3.40
GO:0005496	steroid binding	5.91E-07	2.10E-05	3.54
GO:0043168	anion binding	7.38E-07	2.45E-05	3.22
GO:0036094	small molecule binding	3.29E-06	1.02E-04	2.72
GO:0003708	retinoic acid receptor activity	4.99E-05	1.46E-03	4.08
GO:0042974	retinoic acid receptor binding	9.75E-05	2.70E-03	3.34
GO:0004887	thyroid hormone receptor activity	2.07E-04	5.43E-03	4.08
GO:0008144	drug binding	2.07E-04	5.16E-03	4.08
GO:0046965	retinoid X receptor binding	3.35E-04	7.94E-03	3.27
GO:0005102	receptor binding	4.08E-04	9.24E-03	1.83
**negative Migrants**				
GO:0043565	sequence-specific DNA binding	1.68E-29	8.39E-27	1.43
GO:0003682	chromatin binding	2.03E-05	3.37E-03	1.45
GO:0044877	macromolecular complex binding	3.26E-05	3.25E-03	1.42
GO:0071837	HMG box domain binding	5.38E-05	4.46E-03	2.67
GO:0019904	protein domain specific binding	6.40E-04	3.98E-02	1.66
GO:0001105	RNA polymerase II transcription coactivator act.	9.53E-04	5.27E-02	2.40

The analysis used the 1175 classified TFs as background.

Table S10 - Number of significantly up- or down-regulated TFs at each time point.

	Pioneer		Settler		Migrant+		Migrant-	
Time	Up	Down	Up	Down	Up	Down	Up	Down
00h15m	3	0	2	0	3	0	0	0
00h30m	9	0	5	0	10	0	2	0
00h45m	11	0	11	0	13	0	3	0
01h00m	13	0	11	1	16	0	8	2
01h20m	19	5	14	3	21	1	17	4
01h40m	17	19	11	10	17	10	15	10
02h00m	18	6	13	9	22	6	14	12
02h30m	17	6	13	12	19	7	11	9
03h00m	18	4	15	13	20	8	12	12
03h30m	14	5	15	12	18	9	13	14
04h00m	10	8	12	15	17	13	13	16
05h00m	14	8	18	15	17	15	11	14
06h00m	13	10	9	16	16	12	12	14
07h00m	13	22	13	17	11	16	7	20
08h00m	15	25	15	23	18	29	11	20
12h00m	12	16	11	27	16	18	7	23
16h00m	12	28	10	23	11	29	8	23
24h00m	10	29	9	36	13	35	10	32
42h00m	21	27	14	33	22	41	9	31
60h00m	18	22	15	32	21	29	11	33

Figure S1 - Log ratio of number of up-regulated versus down-regulated TFs.

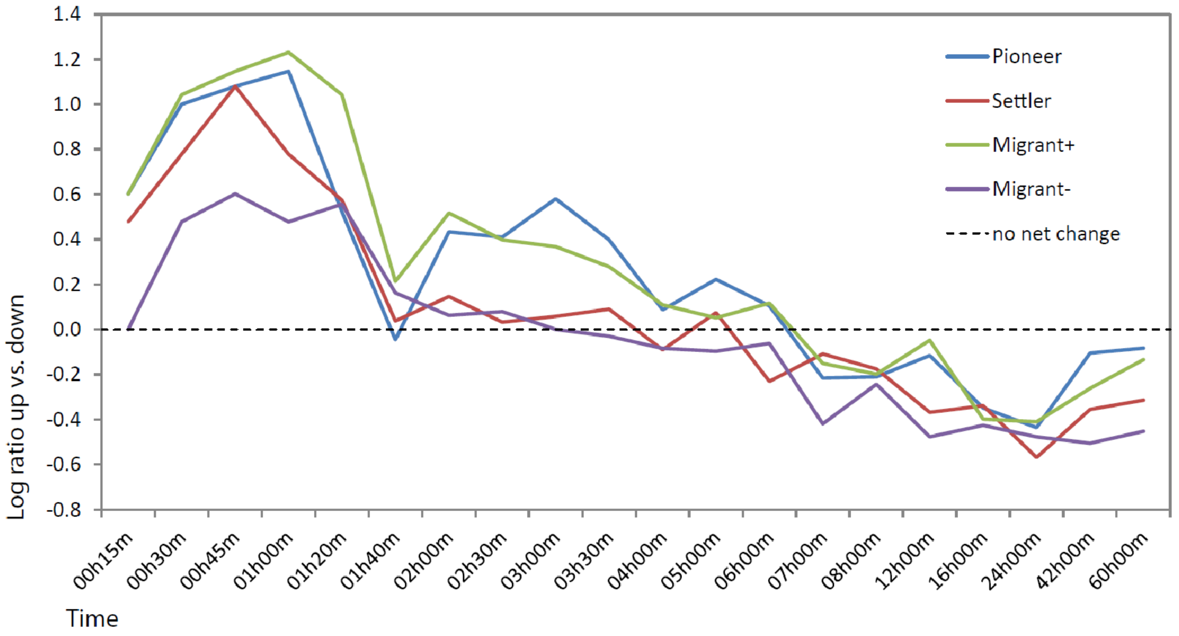

The graph shows the log ratio of number of significantly up-regulated genes versus number of down-regulated genes at each time point for each class of regulatory function. The different classes show similar trends, indicating that they are needed in combination for activation of new genes, at least within the time resolution provided by the experimental data.
